# Relationship between frailty, social support and family functionality of hemodialysis patients: a cross-sectional study

**DOI:** 10.1590/1516-3180.2021.0089.R1.0904221

**Published:** 2021-10-20

**Authors:** Diana Gabriela Mendes dos Santos, Joice Marques Pallone, Carlene Souza Silva Manzini, Marisa Silvana Zazzetta, Fabiana de Souza Orlandi

**Affiliations:** I MD. Master’s Student, Department of Nursing, Universidade Federal de São Carlos (UFSCar), São Carlos (SP), Brazil.; II BSc. Undergraduate Student, Department of Gerontology, Universidade Federal de São Carlos (UFSCar), São Carlos (SP), Brazil.; III PhD. Nurse, Department of Nursing, Universidade Federal de São Carlos (UFSCar), São Carlos (SP), Brazil.; IV MD, PhD. Associate Professor, Department of Gerontology, Universidade Federal de São Carlos (UFSCar), São Carlos (SP), Brazil.; V MD, PhD. Adjunct Professor, Department of Gerontology, Universidade Federal de São Carlos (UFSCar), São Carlos (SP), Brazil.

**Keywords:** Frailty, Renal insufficiency, chronic, Social support, Family relations, Dialyses, Social network, Family support

## Abstract

**BACKGROUND:**

The population with chronic kidney disease (CKD) is more predisposed to early development of frailty. Although the concept of frailty is well established from a physical point of view, it is not an exclusively physical syndrome. It can be characterized as an interaction of physical, psychological and social factors.

**OBJECTIVE:**

To ascertain the relationship between frailty, social support and family functionality among CKD patients undergoing hemodialysis.

**DESIGN AND SETTING:**

Correlational, cross-sectional and quantitative study conducted at a service in the interior of the state of São Paulo.

**METHODS:**

This study included 80 patients with CKD who were on hemodialysis. The participants were interviewed individually, with application of the following instruments: sociodemographic and economic characterization, Tilburg Frailty Indicator, Medical Outcomes Study and Family APGAR. Females and white ethnicity predominated among the participants, and their mean age was 59.63 ± 15.14 years.

**RESULTS:**

There was high prevalence of frailty (93.8%). Although there was a difference in scores for the dimensions of social support between the frail group and the non-frail group, only family functionality reached a statistically relevant difference. There was a significant correlation between physical frailty, social support and family functionality.

**CONCLUSIONS:**

Presence of frailty is related to the social support and family functionality of patients with CKD undergoing hemodialysis.

## INTRODUCTION

According to a Brazilian consensus, frailty consists of a state of physiological age-related vulnerability that is produced through reduction of homeostatic reserves and reduced capacity of the organism in response to negative health outcomes, such as hospital admissions, falls and functional loss, with increased likelihood of death.^1^

Despite being a complex syndrome, frailty can be reversed or mitigated through effective interventions, but for this to occur, it is recommended that screening for frailty should be done early, while patients are still in care.^2^ Although the concept of frailty is well established from the physical point of view, it is not an exclusively physical syndrome. It also encompasses biopsychosocial factors that depend on a holistic view of frail individuals.^3^^,^^4^

The population with chronic kidney disease (CKD) has high incidence and prevalence of physical and cognitive impairment, and is more predisposed to early development of frailty.^5^ In addition, the process of CKD, from diagnosis to treatment with hemodialysis, leads to several biopsychosocial changes in patients’ lives.^6^

Mansur, Damasceno and Bastos carried out a study among 146 patients with CKD undergoing conservative treatment (CT), hemodialysis (HD) or peritoneal dialysis (PD), with the aim of assessing the prevalence of frailty and the factors associated with it.^7^ They pointed out that frailty occurs frequently among patients with CKD undergoing conservative or dialysis treatment, even among those who are not elderly. In addition, a systematic review by Chowdhury et al. showed that frailty was associated with an increased risk of mortality and hospitalization.^8^

In view of this scenario, screening for frailty among patients with CKD is extremely important: not only for elderly individuals but also for younger ones. At early stages of kidney disease, individuals may present frailty that, if untreated, may lead to falls, loss of quality of life, earlier hospitalizations and higher numbers of deaths.

In view of the above, the present study was conducted with the aim of answering the following questions: What is the level of frailty among patients with CKD undergoing hemodialysis? What social characteristics (material support, affective support, emotional support, positive social interaction support, information support and family functionality) are associated with frailty?

## OBJECTIVE

Given the scenario presented, the aim of this study was to evaluate and ascertain the relationship between frailty, social support and family functionality among patients with CKD undergoing hemodialysis.

## METHODS

### Design

This study was characterized as correlational, cross-sectional and quantitative. This investigation was carried out in a renal replacement therapy unit in the municipality of São Carlos, in the interior of the state of São Paulo, Brazil, in 2019.

### Sample

The unit where the study was carried out serves 180 patients. All patients who fulfilled the inclusion criteria (n = 150) were invited to participate in the study and those who accepted made up the sample of the present study, totaling 80 participants. The inclusion criteria were that the subjects needed to have a medical diagnosis of CKD, be under hemodialysis and have preserved oral communication. The exclusion criterion was presentation of dementia, according to the medical records.

### Data collection

The data collection process took place as follows. An initial contact was made with the patients, at which the research was explained and they were invited to participate in the study. Patients who agreed to participate signed a free and informed consent statement. At their next hemodialysis session, and specifically in the first two hours (in which patients present with fewer hemodynamic changes), evaluations were started using a sociodemographic and economic characterization and the Tilburg Frailty Indicator, Medical Outcomes Study Social Support Scale and Family APGAR.

The Tilburg Frailty Indicator (TFI) has the aim of assessing frailty and is considered to be one of the best instruments for this purpose, since it has three dimensions (physical, psychological and social). It was developed by Gobbens et al. in the Netherlands and was adapted for use in Brazil by Santiago et al.^9^^,^^10^ Its scores can range from 0 to 15 points, such that scores < 5 indicate frailty.

The Medical Outcomes Study Social Support Scale was developed by Shernoure and Stewart and was validated for use in Brazil by Andrade in 2001. It has the objective of evaluating social support.^11^^,^^12^ It consists of 19 items that are subdivided into five dimensions of social support: material, affective, emotional, informative and positive social interaction. The total score from this instrument is obtained through scores for each domain ranging from 20 to 100 points. The closer to 100 that the score is, the higher the level of social support is. The responses to each question are scored as follows: never (1), rarely (2), sometimes (3), almost always (4) and always (5). These scores are added together in each dimension. For the Affective Social Support dimension alone, the score obtained should be divided by 15 and then multiplied by 100.^11^

Lastly, the Family APGAR has the aim of ascertaining whether there is any family dysfunction. This instrument was created by Smilkstein^13^ and was adapted for use in Portuguese by Duarte.^14^ It consists of five questions with four answer options: never (0 points), rarely (1 point), sometimes (2 points), almost always (3 points) and always (4 points). The total scores are interpreted as follows: high family dysfunction (1-8 points), moderate family dysfunction (9-12 points) and good family functionality (13-20 points).

### Data analysis

The statistical treatment of the data was performed with the aid of the Statistical Package for the Social Sciences (SPSS) software, version 22.0 (IBM Corporation, Armonk, New York, United States). Descriptive analyses were performed and tables were prepared, containing central trend data (average, minimum and maximum) and dispersion measurements (standard deviation). The Kolmogorov-Smirnov test was performed, which showed that the data did not have normal distribution; hence, nonparametric tests were used.

Spearman’s correlation coefficients were calculated to investigate relationships between continuous variables. The magnitude of correlations was classified as proposed by Levin and Fox (2004): weak (< 0.3); moderate (0.3 to 0.59); strong (0.6 to 0.9); or perfect (1.0).

To compare psychosocial variables, according to the level of frailty measured using the TFI (non-frail or frail), the Mann-Whitney test was used. The significance level adopted for the statistical tests was 5% (P ≤ 0.05).

### Ethical considerations

The protocol for this study was approved by the ethics committee of our institution (CAAE: 18828419.0.0000.5504, number 3.535.236; date: August 27, 2019) and all the subjects signed an informed consent statement.

## RESULTS

This study included 80 patients undergoing hemodialysis treatment. Regarding frailty assessed through the Tilburg Frailty Indicator instrument, 75 of the participants were considered frail. Among these frail patients, the following characteristics were more prevalent: female, white ethnicity, with a partner and retired. The subjects’ average schooling was 6.63 years and they had had 4.51 years of hemodialysis. The most prevalent comorbidity was arterial hypertension, followed by diabetes mellitus (Table 1).

**Table 1.** Sociodemographic categorical variables and economic characteristics. São Carlos (SP), Brazil, 2019 (n = 80)

Variable	Categories	Frail (n = 75)	Non-frail (n = 5)	P-value
Gender	Male	32	4	0.104
	Female	43	1	
Ethnicity	White	48	4	0.502
	Black	20	0	
	Brown	6	1	
	East Asian	1	0	
Marital status	With a fixed partner	48	4	0.760
	No fixed partner	26	1	
Occupation	Retired	52	5	0.708
	Absent^*^	10	0	
	Housewife	10	0	
	Others	2	0	
Comorbidities				
Diabetes	No	45	5	0.074
	Yes	30	0	
Hypertension	No	31	4	0.091
	Yes	44	1	
Other types of Comorbidities	No	68	4	0.441
	Yes	7	1	

^*^Absent from work, as approved by the National Institute of Social Security.

Among the non-frail patients (n = 5), males, white ethnicity and a steady partner were more prevalent. Their average education level was 7.20 years, the average length of time on hemodialysis was 4.76 years and all of them were retired. Among the comorbidities, four individuals were hypertensive (Table 1).

**Table 2.** Sociodemographic continuous variables and economic characteristics. São Carlos (SP), Brazil, 2019 (n = 80)

Variable	Mean	P-value
Age		
Frail (n = 75)	60.00	0.825
Non-frail (n = 5)	59.60	
Education level		
Frail (n = 75)	6.63	0.77
Non-frail (n = 5)	7.20	
Years on hemodialysis		
Frail (n = 75)	4.51	0.58
Non-frail (n = 5)	4.76	

Social support was assessed using the Medical Outcomes Study Social Support Scale. Frail patients obtained lower scores for all dimensions of the Medical Outcomes Study Social Support Scale, in relation to non-frail patients. In spite of this, the averages found were relatively high, using the score range from 20 to 100 points as a parameter. Among the frail patients, the domain with the highest score was Material Support (84.16) and the one with the worst score was Positive Social Interaction Support (72.93). Among the patients considered non-frail, the domain with the highest score was Emotional Support (97.00) and the one with the worst score was Information Support (72.27) (Table 3).

**Table 3.** Descriptive statistics of the Tilburg Frailty Indicator (TFI), Family APGAR and Medical Outcomes Study Social Support Scale (MOS). São Carlos (SP), Brazil, 2019 (n = 80)

Instrument	Category	n	Mean	P-value
MOS				
Material support	Frail	75	84.16	0.278
	Non-frail	5	95.00	
Affective support	Frail	75	78.84	0.146
	Non-frail	5	96.00	
Emotional support	Frail	75	75.47	0.058
	Non-frail	5	97.00	
Positive social interaction support	Frail	75	72.93	0.058
	Non-frail	5	95.00	
Information support	Frail	75	75.27	0.034
	Non-frail	5	96.00	
**APGAR**	Frail	75	13.79	0.004
	Non-frail	5	19.60	

To assess family functionality, the Family APGAR was used. It was found that frail participants scored lower than non-frail participants. Despite this, both groups showed good family functionality. Although there was a difference in scores for the dimensions of the Medical Outcomes Study Social Support Scale between the frail group and the non-frail group, only family functionality reached a statistically relevant difference (Table 3).

In the correlation analyses, frailty showed moderate correlations with material support, affective support, emotional support, positive interaction support, information support and family functionality, all with statistical significance (Table 4).

**Table 4.** Spearman’s correlation of Tilburg Frailty Indicator (TFI) with Material Support (APM), Affective Support (APA), Emotional Support (APE), Positive Social Interaction Support (APISP), Information Support (API) and Family Functionality (APGAR). São Carlos (SP), Brazil, 2019 (n = 80)

		APM	APA	APE	APISP	API	APGAR
**TFI total**	r	-0.485	-0.559	-0.565	-0.481	-0.0543	-0.550
	P-value	**0.008**	**0.003**	**< 0.001**	**< 0.001**	**< 0.001**	**< 0.001**
**Physical dimension**	r	-0.307	-0.262	-0.392	-0.288	-0.366	-0.305
	P-value	**0,006**	**0.019**	**< 0.001**	**0.010**	**< 0.001**	**0.006**
**Psychological dimension**	r	-0.182	-0.220	-0.301	-0.246	-0.303	-0.385
	P-value	**0.006**	**0.019**	**< 0.001**	**0.010**	**< 0.001**	**0.006**
**Social dimension**	r	-0.106	-0.197	-0.226	-0.275	-0.264	-0.142
	P-value	**0.006**	**0.019**	**< 0.001**	**0.010**	**< 0.001**	**0.006**

The physical dimension of the TFI instrument showed negative correlations of moderate magnitude with material support, emotional support, information support and family functionality. The psychological dimension of the TFI instrument presented negative correlations of moderate magnitude with emotional support, information support and family functionality (Table 4).

## DISCUSSION

The sociodemographic characteristics found in the present study have also been pointed out in other investigations that are available in the literature, in Brazil and internationally. One of the characteristics that was distinct between the groups was sex, which was predominantly female in the frail group. According to a study by Fried et al., which included 5,317 elderly patients without CKD, women were more frail than men, regardless of age.^15^

Another study that corroborates our results is the one carried out by Shilipak et al., who compared 5,808 elderly patients with and without CKD and found higher prevalence of frailty among women, regardless of CKD status.^16^ The most prevalent comorbidity in our study was arterial hypertension. This characteristic was also noted in the study by Mansur et al., among 61 patients with CKD who were receiving pre-dialysis treatment, of whom 56.1% had hypertension.^17^

The population with CKD has high incidence and prevalence of physical and cognitive impairment and is more predisposed to early development of frailty, which requires screening before old age is reached.^18^^,^^19^ The high prevalence of frailty found in our study (93.8%) was also seen in a systematic review carried out by Chowdhury et al., in which the prevalence of frailty ranged from 7% among community-dwellers (CKD stages 1-4) to 73% in a cohort of patients on hemodialysis.^8^

Gesualdo conducted a study with the objective of identifying the factors associated with frailty among adults and elderly individuals with CKD who were undergoing hemodialysis. Most of the adults were found to be pre-frail: 54.84% according to Fried’s frailty phenotype; and 58.06% according to the Tilburg Frailty Indicator. Most of the elderly subjects were frail: 64.44% and 73.33%, according to Fried’s frailty phenotype and the TFI, respectively.^5^

This has also been seen in other investigations, such as the cross-sectional study conducted by Bessa, among 191 elderly people who comprised a non-probabilistic sample. In this population, 68.8% were women and the mean age was 75.8 years.^20^ Regarding frailty, 50.0% of the participants were considered frail according to the Tilburg Frailty Indicator. Those findings corroborate the results from the present study, in which frailty was highly prevalent among our patients with CKD undergoing hemodialysis, according to the TFI instrument, comprising 93.8% of the sample.

The psychosocial alterations seen in our study were also observed in a study conducted by Mulasso et al., among 2010 elderly people in an Italian community, who aimed to investigate associations of frailty and psychosocial factors with autonomy in daily activities.^21^ The objectives of their study were to evaluate differences in psychosocial factors between robust, pre-frail and frail individuals, and to investigate whether frailty showed any interactive effect with empirically identified groupings of psychosocial factors, with regard to autonomy in activities of daily living (ADLs). In the results, it was found that 30% of the individuals were robust, 55% pre-frail and 14% frail. Covariance analyses showed that there were differences in all psychosocial variables, in relation to frailty. That study demonstrated the relationship between physical frailty and social frailty and highlighted the importance of psychosocial factors in detecting frailty.

One of the social factors that can impact people’s lifespan is social support. This can be evaluated and classified as perceived support or received support. According to Cramer, Henderson and Scott, perceived support relates to the people that the individual perceives as available in case of need, while received support is social support in the form in which it is received, even when the individuals who provide the support are not identified.^22^

Social support, in turn, is offered by the social network that encompasses the family and neighbors, among others. The ­family plays an essential role in individuals’ social networks, and family rearrangements promote intergenerational coexistence. This experience can contribute positively to the social support received.^23^ Thus, it is understood that each family has functionality and systematics that aim to fulfill and harmonize its essential functions, in a manner appropriate to the identity and tendencies of its members, through acting realistically in relation to the dangers and opportunities that prevail in the social environment.^10^

The association between family functionality and social support relationships is found in other studies, such as Park et al. These authors aimed to evaluate whether loneliness mediated the relationship between social involvement and depressive symptoms and to determine how age moderated the effect of mediation.^24^ The data in this study came from a survey of adults living in the community aged 18 years or older in South Korea, from March to April 2017, in which a total of 1,017 respondents were divided into three age groups (18 to 44, 45 to 64 and 65 years or over). The mediating effect of loneliness was tested with regard to each of three variables relating to social engagement (family network, network of friends and perceived community support) and depressive symptoms. The results showed age-related differences in mediation. The family network had a more pronounced effect in relation to loneliness in the oldest group, while the size of the network of friends significantly predicted loneliness among younger adults. The youngest and oldest groups felt less lonely when they had higher levels of community support; the middle age group was not influenced by the effects of mediation.

This study presented the limitation of selection of the sample by convenience. This makes it difficult to generalize the data. In addition, the imbalance between the frail and non-frail groups made it impossible to carry out logistic regression analyses.

## CONCLUSION

Based on the proposed objectives and the results obtained, it can be concluded that that the presence of frailty was related to social support and family functionality.

Thus, it is important to highlight the need for early screening of frailty in this population. Moreover, there is a need to create public policies that meet the social and psychological demands of these patients, thereby preventing and managing injuries.

From the perspective of expansion of this investigation, longitudinal studies on monitoring the levels of physical and social frailty are desirable. Furthermore, differences in frailty factors between the forms of treatment of CKD should be investigated.

## References

[1] Coelho T, Paúl C, Gobbens RJ, Fernandes L (2015). Determinants of frailty: the added value of assessing medication. Front Aging Neurosci.

[2] Rodríguez-Mañas L, Féart C, Mann G (2013). Searching for an operational definition of frailty: a Delphi method based consensus statement: the frailty operative definition-consensus conference project. J Gerontol A Biol Sci Med Sci.

[3] Gobbens RJ, Van Assen MA (2016). Explaining frailty by lifestyle. Arch Gerontol Geriatr.

[4] Gobbens RJ, Luijkx KG, Wijnen-Sponselee MT, Schols JM (2010). In search of an integral conceptual definition of frailty: opinions of experts. J Am Med Dir Assoc.

[5] Gesualdo GD (2016). Fragilidade de adultos e idosos com doença renal em tratamento hemodialítico: Identificação de fatores associados.

[6] Ibiapina ARS, Soares NSA, Amorin EM (2016). Aspectos psicossociais do paciente renal crônico em terapia hemodialítica. Sanara.

[7] Mansur HN, Damasceno VO, Bastos MG (2012). Prevalência da fragilidade entre os pacientes com doença renal crônica em tratamento conservador e em diálise. J Bras Nefrol.

[8] Chowdhury R, Peel NM, Krosch M, Hubbard RE (2017). Frailty and chronic kidney disease: A systematic review. Arch Gerontol Geriatr.

[9] Gobbens RJ, van Assen MA, Luijkx KG, Wijnen-Sponselee MT, Schols JM (2010). The Tilburg Frailty Indicator: psychometric properties. J Am Med Dir Assoc.

[10] Santiago LM, Luz LL, Mattos IE, Gobbens RJ (2012). Adaptação transcultural do instrumento Tilburg Frailty Indicator (TFI) para a população Brasileira [Cross-cultural adaptation of the Tilburg Frailty Indicator (TFI) for use in the Brazilian population]. Cad Saude Publica.

[11] Sherbourne CD, Stewart AL (1991). The MOS Social Support Survey. Social Science and Medicine.

[12] Andrade CR (2001). Associação entre apoio social e frequência de autoexame das mamas no Estudo Pró-Saúde.

[13] Smilkstein G (1978). The family APGAR: a proposal for a family function test and its use by physicians. J Fam Pract.

[14] Duarte YAO (2001). Família: rede de suporte ou fator estressor - a ótica de idosos e cuidadores familiares.

[15] Fried LP, Tangen CM, Walston J (2001). Frailty in older adults: evidence for a phenotype. J Gerontol A Biol Sci Med Sci.

[16] Shlipak MG, Stehman-Breen C, Fried LF (2004). The presence of frailty in elderly persons with chronic renal insufficiency. Am J Kidney Dis.

[17] Mansur HN, Colugnati FA, Grincenkov FR, Bastos MG (2014). Frailty and quality of life: a cross-sectional study of Brazilian patients with pre-dialysis chronic kidney disease. Health Qual Life Outcomes.

[18] Johansen KL, Chertow GM, Jin C, Kutner NG (2007). Significance of frailty among dialysis patients. J Am Soc Nephrol.

[19] Wilhelm-Leen ER, Hall YN, K Tamura M, Chertow GM (2009). Frailty and chronic kidney disease: the Third National Health and Nutrition Evaluation Survey. Am J Med.

[20] Bessa BML (2016). A Fragilidade Social: um contributo para a compreensão da Síndrome de Fragilidade em pessoas idosas.

[21] Mulasso A, Roppolo M, Giannotta F, Rabaglietti E (2016). Associations of frailty and psychosocial factors with autonomy in daily activities: a cross-sectional study in Italian community-dwelling older adults. Clin Interv Aging.

[22] Cramer D, Henderson S, Scott R (1997). Mental Health and Desired Social Support: A Four-Wave Panel Study. Journal of Social and Personal Relationships.

[23] Duarte MCS, Fernandes MGM, Rodrigues RAP, Nóbrega MML (2013). Prevalência e fatores sociodemográficos associados à fragilidade em mulheres idosas. Rev Bras Enferm.

[24] Park NS, Lee BS, Chiriboga DA, Chung S (2019). Loneliness as a mediator in the relationship between social engagement and depressive symptoms: Age differences among community-dwelling Korean adults. Health Soc Care Community.

